# Thank you, Professor Gattinoni†, and have a good trip to eternity!

**DOI:** 10.62675/2965-2774.20250002

**Published:** 2025-02-21

**Authors:** Guillermo Bugedo, Nicolas Nin, Elisa Estenssoro, Flavia Ribeiro Machado

**Affiliations:** 1 Pontificia Universidad Católica de Chile Departamento de Medicina Intensiva Santiago Chile Departamento de Medicina Intensiva, Pontificia Universidad Católica de Chile -Santiago, Chile.; 2 Unidad de Cuidados Intensivos Hospital Español Juan Jose Crottoggini Montevideo Uruguay Unidad de Cuidados Intensivos, Hospital Español Juan Jose Crottoggini - Montevideo, Uruguay.; 3 Universidad de Montevideo Centro de Ciencias Biomédicas Montevideo Uruguay Centro de Ciencias Biomédicas, Universidad de Montevideo - Montevideo, Uruguay.; 4 Universidad Nacional de La Plata Facultad de Ciencias Médicas La Plata Argentina Facultad de Ciencias Médicas, Universidad Nacional de La Plata - La Plata, Argentina.; 5 Universidade Federal de São Paulo Escola Paulista de Medicina Discipline of Anesthesiology São Paulo SP Brazil Discipline of Anesthesiology, Pain and Intensive Care, Escola Paulista de Medicina, Universidade Federal de São Paulo - São Paulo (SP), Brazil.

At the end of the year, we learned of the death of Professor Luciano Gattinoni (Figure 1), one of the leading researchers in the fields of Critical Care Medicine and acute respiratory failure. For those who work in the intensive care unit, his passing is not a matter of indifference since, together with other collaborators and researchers, he established several pathophysiological principles that guide the ventilatory management of acute respiratory distress syndrome (ARDS). As both a witness and actor in the first stage of modern Critical Care Medicine, during which mechanical ventilation and continuous monitoring of physiological parameters were introduced for the care of critically ill patients, Professor Gattinoni shone with his own light and left an indelible mark on thousands of Critical Care Medicine professionals.

**Figure 1:**
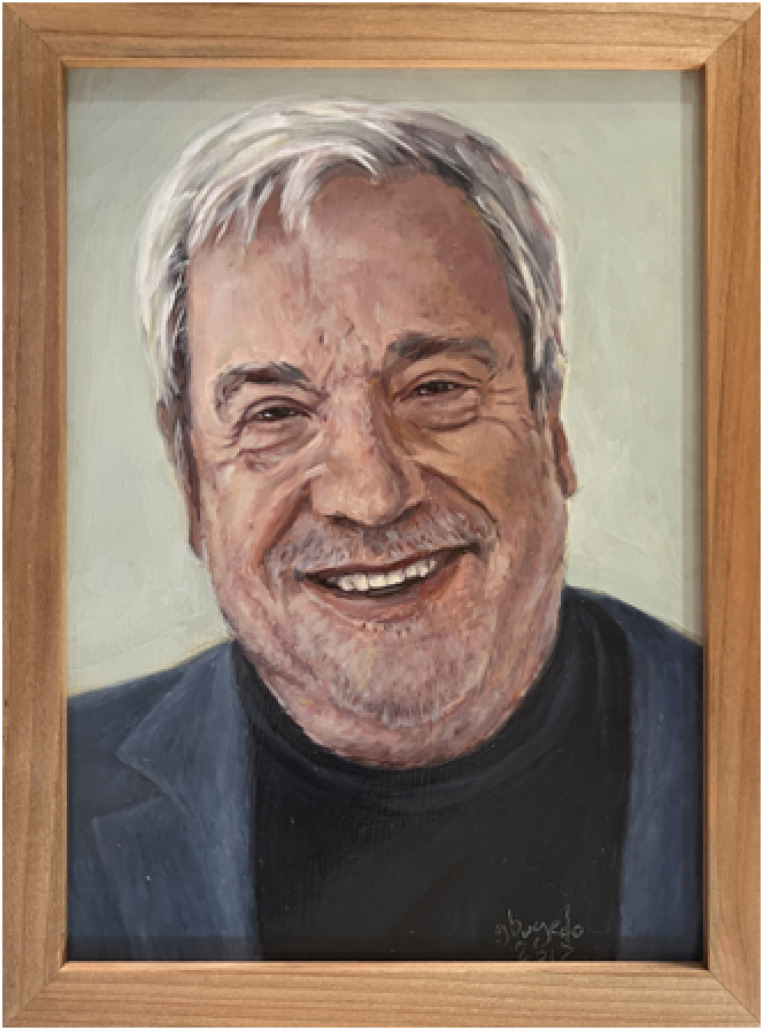
Professor Luciano Gattinoni† (1945–2024).

## Early years, the advent of computed tomography and the description of acute respiratory distress syndrome subphenotypes

Professor Gattinoni began his research career by studying extracorporeal CO_2_ removal as a mechanism to rest the lung during respiratory failure. Luck and effort at the same time, as he himself described, led him to apply these concepts in clinical practice and to obtain surprising results after the failure of the first study of extracorporeal oxygenation (ECMO) in the late 1970s.^(1)^

At that time, computed tomography appeared in clinical practice, which allowed, together with other techniques, visualization of the lung for the first time in three dimensions, both during anesthesia and during respiratory failure.^(2)^ Thus, Gattinoni et al. described for the first time that, in ARDS, the lung is highly heterogeneous and that the deterioration in distensibility tells us of a smaller exchange surface (similar to that of a baby's lung) and not a rigid lung, as previously thought.^(3)^ This baby lung concept led to multiple groups to ventilate these patients with lower tidal volumes until the ARDSnet study was published, which is currently the standard for ventilatory support in ARDS. Professor Gattinoni was also the first to observe that lung mechanics, computed tomography characteristics, and the response to positive end-expiratory pressure and prone positioning greatly differed in patients with pulmonary and extrapulmonary causes of ARDS. These observations led to the first identification of ARDS subphenotypes, which are key to the current understanding of ARDS pathophysiology and for the development of personalized therapeutic approaches in the future.^(4)^

## Sponge lung, prone positioning and clinical trials

Moreover, with the help of a CT scanner, Gattinoni et al. reported that the densities located in the dependent areas of the lung shifted when the patient was in the prone position.^(5)^ Thus, the better perfused areas (dorsal) opened and improved gas exchange, sometimes dramatically. The concept of sponge lung, referring to the dynamic collapse of the dependent areas, was described in an editorial.^(6)^ At the beginning of this century, Professor Gattinoni led the first randomized clinical study of prone positioning in patients with ARDS (Table 1), with encouraging results, and these results were finally confirmed a decade later by Guérin and collaborators. Most likely, many or most patients who required ventilatory support during the swine flu pandemic 15 years ago and recently during the SARS-CoV-2 pandemic recovered because of Professor Gattinoni's scientific contributions.

**Table 1:** Randomized clinical trials led by Luciano Gattinoni

	Intervention	Outcome		
N = 762 critically ill patients*	Three different hemodynamic goals:		ICU-mort	6-month mort
	a) Control group (normal CI: 2.5 and 3.5L/min/m^2^)	Control	48%	62%
	b) Supranormal CI (> 4.5k/min/m^2^)	Supranormal	49%	62%
	c) Venous oxygen-saturation (> 70%) group	SatvO_2_	52%	64%
			p = 0.638	p = 0.875
N = 304 patients with ARDS, and Pa: FiO_2_ < 200 with PEEP 10cmH_2_O†	Two ventilation protocols:		Day-10 mort	6-month mort
	a) Prone for ≥ 6 hours daily for 10 days	Prone	21%	63%
	b) Supine	Supine	25%	59%
			NS	NS
N = 1818 patients with severe sepsis‡	Two fluid replacement solutions:		Day-28 mort	Day-90 mort
	a) 20% albumin and crystalloid solution	20% albumin	32%	41%
	b) Crystalloid solution alone	Crystalloid	32%	44%
			p = 0.94	p = 0.29

Source: Modified from:

* Gattinoni L, Tognoni G, Pesenti A, Taccone P, Mascheroni D, Labarta V, Malacrida R, Di Giulio P, Fumagalli R, Pelosi P, Brazzi L, Latini R; Prone-Supine Study Group. Effect of prone positioning on the survival of patients with acute respiratory failure. N Engl J Med. 2001;345(8):568-73;

† Gattinoni L, Brazzi L, Pelosi P, Latini R, Tognoni G, Pesenti A, et al. A trial of goal-oriented hemodynamic therapy in critically ill patients. SvO2 Collaborative Group. N Engl J Med. 1995;333(16):1025-32;

‡ Caironi P, Tognoni G, Masson S, Fumagalli R, Pesenti A, Romero M, Fanizza C, Caspani L, Faenza S, Grasselli G, Iapichino G, Antonelli M, Parrini V, Fiore G, Latini R, Gattinoni L; ALBIOS Study Investigators. Albumin replacement in patients with severe sepsis or septic shock N Engl J Med. 2014;370(15):1412-21.

CI - cardiac index; ARDS - acute respiratory distress syndrome; PEEP - positive end-expiratory pressure.

Professor Gattinoni expanded his research into various aspects of Critical Care Medicine, particularly during the 1990s, when there was a prevailing belief that optimizing or maximizing oxygen transport could improve outcomes for critically ill patients (Table 1). His groundbreaking study involving 760 patients debunked this notion, and the findings of this study were subsequently validated by other studies (Table 1). In his final major clinical investigation, which included 1,800 patients with sepsis, he demonstrated that albumin is not a universal solution for these patients.

## A comprehensive view of the mechanisms underlying ventilator-induced lung injury

In his quest for an in-depth understanding of the mechanisms by which ventilators cause harm, Professor Gattinoni clearly integrated variables that had generally been discussed separately (tidal volume, pressure, respiratory rate, and flow) in a unique physical entity—mechanical power.^(7)^ Derived from the classic equation of motion, the concept of mechanical power enables calculation of the energy cost per ventilator cycle at the patient's bedside. Increased mechanical power has been repeatedly associated with a worse prognosis; therefore, decreasing mechanical power might become a target to guide safer mechanical ventilation.

## A restless and generous mind

Professor Gattinoni was renowned for his unwavering dedication to mentoring the next generation of critical care specialists. His passion for science and patient care inspired countless clinicians and researchers worldwide, and he left a legacy of excellence and innovation in the field. In Brazil and across Latin America, he frequently participated in meetings and congresses, where he dazzled us with his friendly manner and clarity of concepts. At the turn of the century, the research group in Chile collaborated closely with Professor Gattinoni, and this collaboration sparked scientific debates with the São Paulo team on lung recruitability that were especially passionate and dynamic.^(8)^ After conferences, Professor Gattinoni would stay and share his time with colleagues, often sitting at the piano to play and sing. His music brought people together, creating moments of connection and warmth.

His last contact with Latin America occurred last November during the Congress of the *Associação de Medicina Intensiva Brasileira* (AMIB) in São Paulo. From a large, brilliant screen, he delighted the audience with his concepts of ARDS, mechanical ventilation and ventilator-induced lung injury in his usual clarifying but profound way of explaining everything. When he finished, the audience spontaneously leapt to their feet with a resounding applause. Most of us knew, in that moment, that it was the last goodbye.

It would take days to cover all of Professor Gattinoni's contributions to the field of Intensive Care Medicine. His absence leaves a great void in our community, but his contributions will endure forever within the specialty of Intensive Care Medicine.
